# Supplementary material for: Exploring influences on radiation protection compliance: a directed acyclic graph-based cross-sectional study in a non-teaching hospital in western China
Source: PeerJ. 2025 Oct 13;13:e20083. doi: 10.7717/peerj.20083 (PMC12530198; doi:10.7717/peerj.20083)
Supplement: Supplemental Information 2 [file peerj-13-20083-s002.docx]

Medical staff radiation protection knowledge and practice questionnaire

1. Age:

2. Nationality: 🞎Han 🞎other

3. Gender: 🞎male 🞎female

4.Living mode:🞎Living alone 🞎with parents 🞎 with children 🞎with friends

5.Education level：🞎College and below 🞎Bachelor 🞎≥Master

6.Marital status：🞎Unmarried 🞎Married 🞎Divorced

7.Working unit：🞎Radiology department🞎Department of interventional surgery、🞎Nuclear medicine department🞎Endoscopic center🞎Surgery department🞎Oncology department

8.Career in present unit (years)：

9.Title: 🞎Junior 🞎intermediate 🞎 senior

10.Profession：🞎Doctor 🞎Technicians 🞎Nurse 🞎The other health personnel

11.Number of children：🞎None 🞎1 🞎≥2

12.Experience of radiation protection education 🞎Yes 🞎No

13.Subjective health status：🞎Bad 🞎Average 🞎Good

14.Daily exposure time (hours)： 🞎＜4 🞎 4-8 🞎 ＞8

一、Knowledge (Don't know 0- know 1)

1. I know the difference between ionizing and non-ionizing radiation

🞎Don't know 🞎know

2. I know that the biological effects induced by ionizing radiation are generally

divided into two categories: deterministic effects and stochastic effects

🞎Don't know 🞎know

1. I understand the meaning of the principle of "the lowest possible radiation dose" in radiological examinations

🞎Don't know 🞎know

1. I know the three principles of radiation protection

🞎Don't know 🞎know

1. Do I know how to properly use personal radiation protection equipment

🞎Don't know 🞎know

1. Do I know how to properly use the patient's radiation protection equipment

🞎Don't know 🞎know

1. I am aware of the regulations for radiological workers regarding pregnancy

🞎Don't know 🞎know

1. I know how to consider the differences in radiology between adult and child/adolescent patients.

🞎Don't know 🞎know

1. I understand the meaning of radiation protection culture

🞎Don't know 🞎know

1. I know the meaning of the radiation safety warning sign

🞎Don't know 🞎know

1. Do I know how health checks for radiological workers are organized

🞎Don't know 🞎know

1. I know the procedures for handling ionizing radiation emergencies

🞎Don't know 🞎know

1. I am aware of the dose limits for ionizing radiation for radiologists

🞎Don't know 🞎know

1. I am aware of the public dose limits for ionizing radiation

🞎Don't know 🞎know

二、Attitude (1=strongly disagree; 5=strongly agree)

15. I consider the radiation dose measured by the personal dosimeter to be accurate and reliable

🞎strongly disagree 🞎disagree 🞎General 🞎agree 🞎strongly agree

16.I think the radiation I am exposed to in my daily work is harmful to my health

🞎strongly disagree 🞎disagree 🞎General 🞎agree 🞎strongly agree

17.I believe it is necessary to protect against radiation in the course of daily medical treatment

🞎strongly disagree 🞎disagree 🞎General 🞎agree 🞎strongly agree

18.I think it is necessary to regulate the wearing of protective equipment

🞎strongly disagree 🞎disagree 🞎General 🞎agree 🞎strongly agree

19.I consider it necessary to provide health care workers with regular information and training on radiation protection

🞎strongly disagree 🞎disagree 🞎General 🞎agree 🞎strongly agree

三、Behavior (1=never; 5=always)

20. I consistently wear my personal dosimeter correctly during work

🞎never 🞎seldom 🞎sometimes 🞎often 🞎always

1. I routinely monitor dose readings from my personal dosimeter

🞎never 🞎seldom 🞎sometimes 🞎often 🞎always

1. I properly use radiation protective equipment per protocols

🞎never 🞎seldom 🞎sometimes 🞎often 🞎always

1. I routinely guide patients and families on radiation protection during procedures

🞎never 🞎seldom 🞎sometimes 🞎often 🞎always

1. I actively participate in radiation protection training programs

🞎never 🞎seldom 🞎sometimes 🞎often 🞎always

1. I undergo periodic occupational health examinations

🞎never 🞎seldom 🞎sometimes 🞎often 🞎always

1. I implement the justification principle correctly in my practice

🞎never 🞎seldom 🞎sometimes 🞎often 🞎always
