# Supplementary material for: Exploring influences on radiation protection compliance: a directed acyclic graph-based cross-sectional study in a non-teaching hospital in western China
Source: PeerJ. 2025 Oct 13;13:e20083. doi: 10.7717/peerj.20083 (PMC12530198; doi:10.7717/peerj.20083)
Supplement: Supplemental Information 3 [file peerj-13-20083-s003.docx]

医务人员辐射防护知信行调查问卷

1. 年龄：
2. 民族：🞎汉族 🞎其他
3. 性别:🞎男 🞎女
4. 居住方式：🞎独居 🞎与父母 🞎与子女 🞎与朋友
5. 教育程度：🞎高中及以下 🞎大学本科 🞎硕士 🞎博士及以上
6. 婚姻情况：🞎未婚 🞎已婚 🞎离异 🞎丧偶
7. 科室：🞎放射🞎介入🞎核医学科🞎内镜中心🞎外科🞎手术室🞎肿瘤科
8. 放射工作年限：
9. 职称：🞎初级 🞎中级 🞎高级
10. 工种：🞎医生 🞎技师 🞎护士 🞎物理师 🞎工人
11. 生育情况：🞎否 🞎1个小孩 🞎2个小孩
12. 接受辐射防护的正规培训或课程 🞎≤1次/年 🞎2-3次/年 🞎≥4次/年
13. 主观健康状态：🞎非常差 🞎较差 🞎一般 🞎较好 🞎非常好
14. 每日接触射线时间（小时）：🞎＜4 🞎4-8 🞎＞8
15. 知识维度
16. 我知道电离辐射和非电离辐射的区别

🞎不知道 🞎知道

1. 我知道电离辐射诱发的生物效应，通常分为确定性效应和随机性效应两大类

🞎不知道 🞎知道

1. 我明白在放射检查中“尽可能低的辐射剂量”原则的含义

🞎不知道 🞎知道

1. 我知道辐射防护的三大原则

🞎不知道 🞎知道

1. 我知道如何正确使用个人辐射防护设备

🞎不知道 🞎知道

1. 我知道如何正确使用病人的辐射防护设备

🞎不知道 🞎知道

1. 我知道有关怀孕的放射工作人员相关规定

🞎不知道 🞎知道

1. 我知道如何考虑成人和儿童/青少年患者在放射学检查中的差异

🞎不知道 🞎知道

1. 我了解辐射防护文化的含义

🞎不知道 🞎知道

1. 我知道有关辐射安全警示标志的含义

🞎不知道 🞎知道

1. 我知道放射工作人员的健康体检是如何组织起来的

🞎不知道 🞎知道

1. 我知道电离辐射突发事件应急处置相关流程

🞎不知道 🞎知道

1. 我知道放射工作人员电离辐射的剂量限值

🞎不知道 🞎知道

1. 我知道公众电离辐射的剂量限值

🞎不知道 🞎知道

二、态度维度

1. 我认为个人剂量计测得的辐射剂量准确可靠

🞎非常不同意 🞎不同意 🞎一般 🞎不同意 🞎非常同意

1. 我认为在日常工作中接触的射线会危害健康

🞎非常不同意 🞎不同意 🞎一般 🞎不同意 🞎非常同意

1. 我认为有必要在日常医疗诊疗过程中做好辐射防护

🞎非常不同意 🞎不同意 🞎一般 🞎不同意 🞎非常同意

1. 我认为有必要规范穿戴防护用品

🞎非常不同意 🞎不同意 🞎一般 🞎不同意 🞎非常同意

1. 我认为有必要定期向医护人员提供辐射防护的知识和培训

🞎非常不同意 🞎不同意 🞎一般 🞎不同意 🞎非常同意

三、信念维度

1. 我会在工作时正确佩戴个人剂量计

🞎从不 🞎偶尔 🞎有时 🞎经常 🞎总是

1. 我会关注个人剂量计的剂量监测结果

🞎从不 🞎偶尔 🞎有时 🞎经常 🞎总是

1. 我会按规定正确穿戴辐射防护安全设备

🞎从不 🞎偶尔 🞎有时 🞎经常 🞎总是

1. 我会在工作中规范指导患者及家属做好防护

🞎从不 🞎偶尔 🞎有时 🞎经常 🞎总是

1. 我会积极参加辐射防护的相关培训

🞎从不 🞎偶尔 🞎有时 🞎经常 🞎总是

1. 我会定期进行职业健康检查

🞎从不 🞎偶尔 🞎有时 🞎经常 🞎总是

1. 我在工作中会正确践行“正当性原则”

🞎从不 🞎偶尔 🞎有时 🞎经常 🞎总是
